# Do Implicit Attitudes Predict Actual Voting Behavior Particularly for Undecided Voters?

**DOI:** 10.1371/journal.pone.0044130

**Published:** 2012-08-29

**Authors:** Malte Friese, Colin Tucker Smith, Thomas Plischke, Matthias Bluemke, Brian A. Nosek

**Affiliations:** 1 Department of Psychology, University of Basel, Basel, Switzerland; 2 Department of Psychology, University of Florida, Gainesville, Florida, United States of America; 3 Department of Political Science, University of Mannheim, Mannheim, Germany; 4 Department of Psychology, University of Heidelberg, Heidelberg, Germany; 5 Department of Psychology, University of Virginia, Charlottesville, Virginia, United States of America; George Mason University/Krasnow Institute for Advanced Study, United States of America

## Abstract

The prediction of voting behavior of undecided voters poses a challenge to psychologists and pollsters. Recently, researchers argued that implicit attitudes would predict voting behavior particularly for undecided voters whereas explicit attitudes would predict voting behavior particularly for decided voters. We tested this assumption in two studies in two countries with distinct political systems in the context of real political elections. Results revealed that (a) explicit attitudes predicted voting behavior better than implicit attitudes for both decided and undecided voters, and (b) implicit attitudes predicted voting behavior better for decided than undecided voters. We propose that greater elaboration of attitudes produces stronger convergence between implicit and explicit attitudes resulting in better predictive validity of both, and less *incremental* validity of implicit over explicit attitudes for the prediction of voting behavior. However, greater incremental predictive validity of implicit over explicit attitudes may be associated with less elaboration.

## Introduction

Reporting an intention to perform a behavior is an excellent predictor of ultimately performing that behavior [1]. As a consequence, reported behavioral intentions are central measurement for survey research applications. A large research literature provides evidence that behavior intentions can also fail to predict behavior [2], [3] – particularly for extinguishing undesired behaviors (e.g., smoking) or initiating desired ones (e.g., exercise). But there is, as yet, little knowledge about the opposite situation – how to predict behavior when the respondent is unwilling or unable to report a behavioral intention in the first place?

Voting is probably the most prominent behavior for which this question has direct relevance. When asked to indicate their voting plans for an upcoming election, a sizable portion of the electorate reports that they have not yet decided. Predicting the voting behavior of these ‘undecided’ voters has been an unsolved challenge for pollsters and psychologists for many years [4]. Because undecided voters can remain a sizable fraction of voters even days prior to the election [5] they often disrupt the accuracy of polls for predicting election outcomes. How can psychologists and pollsters predict the voting behavior of undecided voters?

In recent years, psychologists offered an interesting answer to this question [6]–[8]. While undecided voters may not yet have developed a behavioral intention, they may possess attitudes that ultimately predict their behavioral intention and vote. Attitudes – feelings of favor or disfavor for social objects – are understood to be important predictors of behavior [9]. Modern conceptions of attitudes propose two types [10]–[12] – explicit attitudes that reflect evaluations that are introspectively identified, deliberately reported, and endorsed by the respondent; and, implicit attitudes that reflect evaluations that are assessed indirectly and may indicate associations of which the respondent is less likely to be aware, less able to control, and may not endorse if given the opportunity.

There is some debate in the literature about whether implicit and explicit attitudes are really distinct constructs, or whether implicit and explicit measures simply assess the same underlying construct in different ways [13]–[15]. There is also debate about to what extent different implicit measures assess automatic processes and which characteristics of automaticity they meet [16]. While we cannot resolve these debates in the current manuscript, we use the most commonly terminology of implicit and explicit attitudes, and refer the reader to these discussions.

Galdi and colleagues (2008) proposed that explicit attitudes will not be useful in predicting the vote for undecided voters, but that implicit attitudes predispose undecided voters to later vote for a particular candidate or party, even if they are unable or unwilling to report a voting intention. By contrast, explicit, but not implicit attitudes are assumed to predict voting behavior for decided voters who are able to report a voting intention. In a study testing this idea [7], residents of a city in northern Italy completed a Single Category Implicit Association Test (SC-IAT) [17], [18] assessing implicit attitudes and self-report questionnaires assessing explicit attitudes toward the enlargement of a local U.S. military base. In addition, they indicated whether they were in favor, undecided, or against the enlargement. One week later, participants completed the measures a second time. Note that Galdi et al. [7] assessed participants’ *opinions* on the issue, not actual voting behavior or voting intentions. In a multiple regression with explicit and implicit attitudes as predictors, explicit, but not implicit, attitudes (measured at time 1) strongly predicted participants’ opinions on the enlargement for *decided* individuals at time 2. A similar regression for *undecided* individuals showed the opposite pattern; implicit, but not explicit, attitudes predicted participants’ opinions at time 2. Although not tested directly, the authors concluded that implicit measures can significantly enhance the prediction of political election outcomes, especially for undecided voters.

These results are an intriguing addition to a large, and rapidly growing, literature examining the role of implicit and explicit attitudes independently and jointly predicting behavior [19]–[21]. If these results would hold in the context of real political elections this would represent a major advancement in the prediction of political election forecasts with significant applied implications. However, the evidence for this assumption heretofore is very intriguing, but not very strong: The data by Galdi and colleagues were obtained in a single study in the context of a nonbinding opinion poll on a specific issue of local politics. There could be important differences in the psychological processes underlying decision making in the context of a specific issue of limited scope and duration as opposed to casting a vote in major elections that are known to be heavily influenced by political attitudes acquired over the lifespan [22].

Even though the study by Galdi and colleagues [7] was conducted in the context of a nonbinding opinion poll on a specific issue of local politics, it gained a lot of its impact through the assumption that the findings would directly generalize to the context of actual political voting behavior in major political elections [7], [8]. Hence, the present research is a test of claims that were derived from this work. We did *not* seek to directly replicate their work and we do not question the results obtained by Galdi et al. [7]. Instead, we sought to evaluate the following four claims:

1. Implicit attitudes predict voting behavior better than explicit attitudes for undecided voters.
2. Explicit attitudes predict voting behavior better than implicit attitudes for decided voters.
3. Implicit attitudes predict voting behavior better for undecided than decided voters.
4. Explicit attitudes predict voting behavior better for decided than undecided voters. Galdi et al. (2008) expected

[…] that future choices of undecided individuals can be predicted by their current automatic mental associations, even when these individuals consciously report that they are still undecided. This case is contrasted with future choices made by decided individuals, which we expected to be guided by consciously held beliefs about choice options rather than automatic mental associations. (p. 1100)

These hypotheses resemble the first and second claim, which indeed describe the result pattern obtained by Galdi et al. [7]. We sought to establish this pattern in the context of political elections involving actual voting behavior. The hypotheses by Galdi and colleagues further imply claims 3 and 4, even though they only found support for the claim that implicit attitudes predict participants’ later opinions better for undecided than decided participants (claim 3), but not for the claim that explicit attitudes predict participants’ later opinions better for decided than undecided participants (claim 4, see note 19 on page 1102).

Based on the extant research literature, the political domain is a challenging area for implicit attitudes to show incremental predictive validity as political cognitions tend to be well-elaborated, have clear, and opposing, positions, and be socially acceptable to share publicly – all of which suggest a decided predictive advantage for explicit attitudes [23]. So, the fact that it is such a highly controllable and deliberate act would seem to leave little room for incremental prediction by implicit attitudes [24], [25]. To reflect the pattern of results obtained by Galdi et al. [7] this general trend would have to be strongly reversed for undecided voters.

We investigated self-reports of voting behavior in national political elections in two different countries with distinct political systems. Study 1 investigated voting behavior in the context of the 2008 presidential election in the U.S., and Study 2 was concerned with the 2009 national parliamentary election in Germany.

## Study 1

Study 1 took place in the run-up of the U.S. presidential election in 2008. In the center of this election campaign was the highly personalized duel between John McCain (Republican) and Barack Obama (Democratic).

### Methods

#### Ethics statement

The University of Virginia Institutional Review Board (IRB) for the Social and Behavioral Sciences approved this research and informed consent process (#2002-0232). Participants were given written informed consent prior to participation, and received a written debriefing at the end of each study session.

#### Participants

Participants were volunteers at the Project Implicit website (https://implicit.harvard.edu/). Of 8784 U.S. citizens reporting to be eligible to vote who completed at least the implicit and explicit measures in the first session, 3884 returned and reported whom they had voted for within 90 days after the election (return rate of 44.22%, 65.20% females, 34.50% males, 0.30% did not report their gender). Mean age was 36.73 years (*SD* = 14.55).

#### Procedure

Participants could take part in up to four sessions, three pre-election sessions and one post-election survey. Only the first pre-election survey (assessed between July 20th and October 19th, 2008) and the post-election survey (assessed directly after the election) are relevant for present purposes.

In session 1, participants completed a demographics questionnaire, an implicit measure assessing relative preference for McCain compared to Obama, and explicit measures of voting intentions, attitudes, and other items that are not relevant for present purposes. The order of these three parts was randomized. Immediately after polling places had closed on November 4, 2008, participants were sent an email asking for whom they had voted. On average, participants took part in the first part of the study 52.88 days (*SD* = 23.57) before the election and indicated their voting behavior 6.94 days (*SD* = 8.73) after the election.

#### Measures

We used an Implicit Association Test (IAT) [26] to assess implicit attitudes toward McCain and Obama following the procedure outlined in Table 1. Each category was represented by five stimuli. Stimuli of the candidates were head-only pictures. Evaluative stimuli were positive and negative words (e.g., peace, laughter, agony, hurt). Evaluative category labels and stimuli were presented in green color, candidates’ category labels in white color. The trials alternated between evaluative and candidate items. The order of combined blocks (i.e. Obama/Good and McCain/Bad vs. Obama/Bad and McCain/Good) was randomized across participants. IAT scores were calculated using the D1 algorithm [27] such that more positive scores indicate a more positive implicit attitude toward Obama compared to McCain. Spearman-Brown corrected split-half reliability of the IAT at time 1, calculated as the correlation between the IAT *D* of blocks 3 and 6 with the IAT *D* of blocks 4 and 7, was *r* = .79. The IAT reliability for decided voters was *r* = .79, and for undecided voters was *r* = .66.

**Table 1:** Procedure of IATs in Studies 1 and 2.

Study	Block	Category labels for leftresponse key	Category labels for rightresponse key	No. of Trials	IAT (only Study 2)
1	1	John McCain	Barack Obama	20	
	2	Good	Bad	20	
	3	John McCain & Good	Barack Obama & Bad	20	
	4	John McCain & Good	Barack Obama & Bad	40	
	5	Barack Obama	John McCain	40	
	6	Barack Obama & Good	John McCain & Bad	20	
	7	Barack Obama & Good	John McCain & Bad	40	
2	1	SPD/Green	CDU/FDP	20	Camps
	2	Good	Bad	20	Camps
	3	SPD/Green & Good	CDU/FDP & Bad	40	Camps
	4	Bad	Good	40	Camps
	5	SPD/Green & Bad	CDU/FDP & Good	40	Camps
	6	Steinmeier & Bad	Merkel & Good	40	Candidates
	7	Good	Bad	40	Candidates
	8	Steinmeier & Good	Merkel & Bad	40	Candidates

*Note*. IAT  =  implicit association test. The order of combined blocks was experimentally controlled across participants.

Explicit attitudes were assessed with the following question: “Please rate how warm or cold you feel toward the following presidential candidates (0 = coldest feelings, 5 = neutral, 10 = warmest feelings)”. This question was followed by two drop-down menus, one for McCain and one for Obama. The difference between the two ratings served as the indicator of explicit attitudes with higher scores indicating a preference for Obama over McCain.

Voting intention was assessed with the following question: “When the United States presidential election is held in November 2008, which of the following candidates do you plan to vote for?” Response options were ‘I have not yet decided’, ‘I will vote for John McCain (R)’, ‘I will vote for Barack Obama (D)’, ‘I will vote for another candidate’, ‘I plan not to vote’, and ‘I will not be eligible to vote’. Participants who indicated that they had not yet decided were classified as undecided (0). The remaining participants eligible to vote were classified as decided (1). Including participants who had planned not to vote but ended up doing so into the group of undecided voters (*n* = 8, 0.2% of the final sample) did not appreciably alter the results.

Voting behavior was assessed with the following question: “Please click on the link that accurately summarizes your vote or non-vote in the 2008 presidential election” accompanied with four hyperlinks named ‘I did not vote in the 2008 presidential election’, ‘I voted for Obama/Biden’, ‘I voted for McCain/Palin’, and ‘I voted for a third-party candidate’. Votes for McCain (Obama) were coded as 0 (1). All other (non-)votes were discarded for the main analyses.

### Results and Discussion

#### Preliminary analyses

All continuous variables were z-standardized before running the logistic regression analyses [28]. Participants who completed more than 10% of their trials in less than 300 ms (0.14%) or with more than 25% (0.96%) errors in the IAT were excluded from data analyses, leading to a final sample of 3594 voters (303 undecided, 8.4%). The time span between the first measurement and the election was larger for undecided (*M* = 56.66, *SD* = 22.71) as compared to decided participants (*M* = 52.49, *SD* = 23.57; *t*(3592) = 2.95, *p* = .003, Cohen’s *d* = 0.18). This is expected as the number of undecided voters tends to decrease as the election approaches. Controlling for this time span in the joint analyses of decided and undecided voters did not appreciably change the results. This is true for both entering time span as a covariate and as a full factor including all two-way and three-way interactions. These analyses are included in the supplementary online material (Table S1). Implicit-explicit correspondence was high (*r* = .62), and higher among decided (*r* = .62) as compared to undecided voters (*r* = .45; *z* = 4.07, *p*s of correlations and their respective difference <.001). This may be a function of differences in the degree of cognitive elaboration of political attitudes between decided and undecided voters, a moderator of implicit-explicit correspondence [24], [29], [30].

#### Main analyses

We first investigated the claims that implicit attitudes predict voting behavior better than explicit attitudes for undecided voters and explicit attitudes predict voting behavior better than implicit attitudes for decided voters (claims 1 and 2). Table 2 shows two distinct models at step 1– step 1a with implicit as the predictor, step 1b with explicit as the predictor – and then step 2 presents the two predictors simultaneously. These show that both implicit and explicit attitudes predicted voting outcome, and that a substantial portion of that predictive validity was in the shared variance between them. Implicit attitudes increased Nagelkerke’s *R* ^2^ by 0.6 and 2.5 percentage points for decided and undecided voters, respectively, after accounting for explicit attitudes and increased the percentage of correctly classified cases by 0.1 and 0.0 percentage points for decided and undecided voters. Explicit attitudes increased Nagelkerke’s *R* ^2^ by 31.5 and 29.5 percentage points for decided and undecided voters, respectively, after accounting for implicit attitudes. Correctly classified cases increased by 7.2 percentage points for decided and by 10.6 percentage points for undecided voters. Thus, the explicit measure predicted voting behavior better than the implicit measure for both decided and undecided voters, corroborating claim 2 and at odds with claim 1.

**Table 2:** Results of the multiple binary logistic regression analyses in Study 1, separately for decided and undecided voters.

Step	Variable	B	*SE*	Wald	*p*	Exp(B)	Nagel-kerke’s R^2^	% CCC
Decided voters (*N* = 3291)								
1a	Constant	3.097	.110	787.694	<.001	22.140	.575	91.2
	IAT	2.392	.099	581.315	<.001	10.932		
1b	Constant	4.874	.244	397.666	<.001	130.813	.884	98.3
	Explicit	4.582	.236	377.262	<.001	97.661		
2	Constant	4.861	.248	383.795	<.001	129.107	.890	98.4
	IAT	.741	.170	19.061	<.001	2.098		
	Explicit	4.061	.244	277.361	<.001	58.024		
Undecided voters (*N* = 303)								
1a	Constant	1.663	.205	65.493	<.001	5.275	.226	71.6
	IAT	1.178	.183	41.271	<.001	3.247		
1b	Constant	3.647	.393	86.207	<.001	38.370	.496	82.2
	Explicit	3.299	.389	72.038	<.001	27.099		
2	Constant	3.791	.408	86.157	<.001	44.280	.521	82.2
	IAT	.622	.221	7.942	.005	1.862		
	Explicit	2.949	.399	54.656	<.001	19.083		

*Note*. B: regression weight B; *SE*: standard error of the regression weight B; Wald: Wald criterion; Exp(B): Odds ratio. Relative amount by which the odds increase (Exp(B)>1.0) or decrease (Exp(B)<1.0) when the value of the predictor is increased by 1 unit; CCC: correctly classified cases; DV: voting behavior (0 = McCain, 1 = Obama). The IAT, explicit measure and decidedness information used in this analysis was obtained at time 1. All continuous variables were z-standardized separately for decided and undecided voters prior to the analyses.

**Figure 1:**
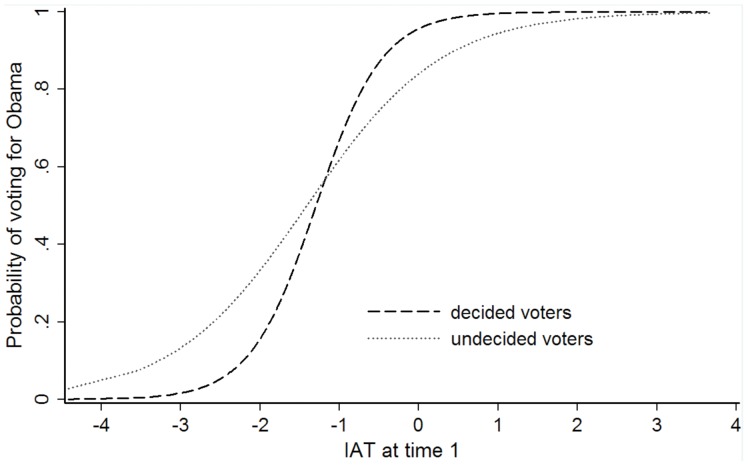
Probability of voting for Obama (vs. McCain). Probability of voting for Obama (vs. McCain) as a function of IAT, decidedness, and their interaction at time 1. High values indicate stronger implicit preferences for Obama (relative to McCain), and a higher probability of voting for Obama (vs. McCain). The IAT predicted the dichotomous choice of vote better for decided as compared to undecided voters as indicated by the steeper line for decided as compared to undecided voters. This indicates that the region of unclear prediction on the basis of IAT-scores (abscissa) between voting for McCain (score on the ordinate of 0) and voting for Obama (score on the ordinate of 1) was smaller for decided than undecided voters, leading to more correctly predicted votes for decided than undecided individuals. IAT scores were z-standardized prior to the analysis.

Next, we investigated the claims that irrespective of explicit attitudes, implicit attitudes predicted voting behavior better for undecided than decided voters, and that irrespective of implicit attitudes, explicit attitudes predicted voting behavior better for decided than undecided participants (claims 3 and 4). Results of the joint multiple binary logistic regression analyses for decided and undecided voters are depicted in Table 3. In a first step, the IAT predicted voting behavior (Nagelkerke’s *R* ^2^ = .538), correctly classifying 89.5% of the participants’ eventual votes. And, in step 2, decidedness moderated the IAT’s influence on voting behavior. However, the moderation was in the opposite direction of the results reported by Galdi et al. [7]. The IAT was a better predictor of voting behavior for *decided* as compared to undecided voters (see Figure 1). This interaction remained descriptively in the same direction, but was not statistically significant anymore after explicit attitudes were included in the model (step 3). Adding explicit attitudes increased Nagelkerke’s *R* ^2^ to.852 and the correct classification of vote to 97.1%. Further, implicit attitudes remained a significant but weak predictor of voting after removing its shared variance with explicit attitudes. In the final model including all two-way interactions (step 4), the IAT × decidedness interaction remained non-significant and the explicit measure was more powerful in the prediction of voting behavior for decided voters.

**Table 3:** Results of multiple binary logistic regression analyses in Study 1 including both decided and undecided voters.

Step	Variable	B	*SE*	Wald	*p*	Exp(B)	Nagel-kerke’s R^2^	% CCC
1	Constant	2.889	.096	914.479	<.001	17.978	.538	89.5
	IAT	2.211	.086	664.147	<.001	9.123		
2	Constant	1.663	.205	65.493	<.001	5.275	.549	89.5
	IAT	1.178	.183	41.271	<.001	3.247		
	Decidedness	1.434	.233	37.824	<.001	4.197		
	IAT* Decidedness	1.214	.208	33.906	<.001	3.366		
3	Constant	4.522	.310	213.234	<.001	92.051	.852	97.1
	IAT	.538	.234	5.305	.021	1.713		
	Decidedness	.155	.305	.258	.612	1.167		
	IAT* Decidedness	.254	.282	.813	.367	1.289		
	Explicit	3.812	.202	355.389	<.001	45.226		
4	Constant	3.333	.405	67.852	<.001	28.029	.856	97.1
	IAT	−.074	.274	.062	.804	.929		
	Decidedness	1.363	.467	8.512	.004	3.909		
	IAT* Decidedness	.164	.285	.329	.566	1.178		
	Explicit	2.338	.429	29.710	<.001	10.357		
	Explicit* Decidedness	1.349	.479	7.951	.005	3.855		
	IAT* Explicit	−.827	.243	11.596	.001	.438		

*Note*. *N* = 3594. B: regression weight B; *SE*: standard error of the regression weight B; Wald: Wald criterion; Exp(B): Odds ratio. Relative amount by which the odds increase (Exp(B)>1.0) or decrease (Exp(B)<1.0) when the value of the predictor is increased by 1 unit; CCC: correctly classified cases; DV: voting behavior (0 = McCain, 1 = Obama). The IAT, explicit measure and decidedness information used in this analysis was obtained at time 1. All continuous variables were z-standardized prior to the analyses.

In sum, these data corroborate the findings of Galdi et al. [7] partly, but not completely. On their own, explicit attitudes were a better predictor of vote than implicit attitudes for both decided and undecided voters. Implicit attitudes predicted voting behavior as well, but showed little predictive validity for either undecided or decided voters that was not accounted for by explicit attitudes. More dramatically, opposite to Galdi and colleagues’ findings, implicit attitudes predicted voting behavior better for *decided* as compared to *undecided* individuals. The present finding is consistent with a meta-analysis [19] suggesting that stronger implicit-explicit relations are associated with better predictive validity for both implicit and explicit attitudes, a topic that will be addressed again in more detail in the general discussion.

## Study 2

Given the striking differences between our data and that by Galdi and colleagues [7], we sought to replicate the results of Study 1 in a different country with a different political system. The study took place in the run-up to the 2009 parliamentary election in Germany (“Bundestagswahl”). Converging evidence with Study 1 would be particularly convincing, because several characteristics of this election were different from the 2008 U.S. presidential election. First, while in the U.S. presidential election voters cast their vote for Barack Obama or John McCain (or one of the far less prominent alternative candidates), in Germany the political parties play a much bigger role. In a somewhat complex voting system, voters cast two votes, the most important in terms of designating the future chancellor (head of government) is a vote for a political party, not a particular person. Most of the time, no single party will attract enough votes to be able to elect the chancellor on its own. Instead, at least one other party is needed to form a coalition. While the right-wing parties CDU/CSU and FDP are predisposed to form a coalition commonly known as ‘black-yellow’ (‘schwarz-gelb’, based on the respective identifying party colors), the left-wing parties SPD and Greens are predisposed to form a coalition commonly known as ‘red-green’ (‘rot-grün’). The fifth and last party expected to enter the parliament was another left-wing party (The Left) that the other major parties had a priori declared that they would not include in their potential coalition.

Although political parties assume a bigger role in German parliamentary elections than in the U.S., elections are nevertheless increasingly candidate-oriented in the sense that voters vote for a particular party with the ultimate goal to support a particular candidate for chancellor. To make allowance for this development, in Study 2 all participants completed both a ‘political-camps IAT’ featuring the prominent ‘black-yellow’ and ‘red-green’ coalitions and a ‘candidates IAT’ featuring the two candidates for chancellor, incumbent chancellor Angela Merkel (CDU) and her contender, Frank-Walter Steinmeier (SPD).

In addition to replicating the results in a different political system, we sought to extend Study 1 in two ways: First, we added a more stringent test of implicit attitudes’ incremental validity beyond explicit attitudes. Even though incremental validity of implicit attitudes was weak in Study 1, it was significant in several analyses. In Study 1, we used a single difference score item as indicator of explicit attitudes that was constructed to be parallel to the IAT. The strong predictive validity of this single item suggests that even though undecided participants were unwilling or unable to commit to a voting intention, explicit attitudes were a good indicator of which political candidate they were leaning to. In supplementary analyses of Study 2 we included a second indicator of explicit attitudes based on separate explicit evaluations of the five major parties to more stringently test if implicit attitudes can add unique predictive value that cannot be accounted for by a more thorough assessment of explicit attitudes.

Second, we sought to test a further hypothesis drawn from the work by Galdi and colleagues [7], [31]. These authors investigated a possible psychological mechanism underlying their finding of differential prediction of decided and undecided voters behavior with explicit and implicit attitudes. They used a two-wave-two-variable panel design to examine implicit and explicit attitude change as a function of decidedness during the time between the two measurement occasions. Results revealed that explicit attitudes predicted later implicit attitudes in *decided* individuals while implicit attitudes had no effect on later explicit attitudes. By contrast, implicit attitudes predicted later explicit attitudes in *undecided* individuals while explicit attitudes had no effect on later implicit attitudes. These results suggest that implicit and explicit attitudes influenced each other in different ways as a function of decidedness [32], [33], and that these changes may have impacted the later expression of opinion. Study 2 allowed us to conceptually replicate this analysis in the context of an actual political election.

### Methods

#### Ethics statement

The study was conducted online. Participants could drop out of the study at any time without any negative consequences. On the first page, participants received brief information about the duration of the study, the measures involved (implicit association tests and questionnaires about politicians, political parties, and the 2009 parliamentary elections in Germany), the involved researchers, their affiliations, and contact information. In addition, they received information about the remuneration for their participation. All data was analyzed anonymously. At the leading institution (Department of Psychology, University of Basel) there was no legal requirement to obtain approval from an IRB for non-clinical research studies, and there in fact was no IRB at the university at the time the study was conducted. An external IRB of the state focuses on clinical, biological, and neuroscientific studies.

#### Participants

We recruited participants for an online study using two different channels, the German Longitudinal Election Study (GLES) [34] and advertisements on the Internet (e.g., postings on relevant websites, Google Adwords). In the former subsample, we were able to oversample undecided voters to make sure that a sufficient number of undecided voters would enter data analysis (39% in the final subsample versus 18% in the final subsample of the Internet-recruited subsample).

Of 1220 eligible voters who provided data for at least the first IAT and the corresponding explicit measure in the initial data collection, 913 responded to the post-election survey and indicated which party they had voted for in the election (return rate of 74.8%). Gender was distributed roughly equally (49.7% females) and the mean age was 39 years (*SD* = 14.32). If they wished, participants entered a lottery of a mobile music player and ten vouchers for a popular online store of music, books, and other products (€15 each). In addition, they could request a report of aggregated study results that was later sent to them.

#### Procedure

Respondents of the GLES were asked at the end of the survey whether they would be willing to participate in a related online study. If they agreed, they received an invitation and a link to the study via email. Participants in the Internet-recruited subsample clicked on a link to reach the study.

The study started with the ‘political camps IAT’ and the ‘candidates IAT’ in a fixed order, followed by measures of explicit attitudes, voting intention, and control questions including demographic data. After the election, participants received an invitation to complete the second part of the study, in which they indicated which party they had voted for in the election, and completed the measures of explicit attitudes, control questions, and demographics again. In addition, we asked participants to complete the ‘political camps IAT’ again on a voluntary basis (552 did so in total, 186 of which were undecided at time 1). On average, participants took part in the first part of the study 51.74 days (*SD* = 44.94) before, and in the second part 2.48 days (*SD* = 1.85) after the election.

#### Measures

The IAT assessments followed the procedure outlined in Table 1. Each category was represented by five stimuli. The political camps were represented by the two party logos, pictures of the most prominent representative of each party, and the coalition name. Political candidates were represented by four head-only pictures and a verbal stimulus depicting the respective candidate name. Evaluative stimuli were positive and negative words (e.g., love, fun, fear, hatred). Evaluative category labels and stimuli were presented in blue color, coalition/candidate’s labels and stimuli in white color. The trials alternated between evaluative and coalition/candidate items. The order of the combined blocks in the political camps IAT was counterbalanced across participants and matched the first combined block of the ‘candidates IAT’ in the sense that *CDU/FDP* was replaced by *Merkel* and *SPD/Green* was replaced by *Steinmeier*. We kept the response key assignment of the political parties constant (CDU/FDP always right, SPD/Green always left) in order to avoid confusion about the political and the spatial meaning of the concepts ‘left’ and ‘right’. IAT scores were calculated using the D1 algorithm [27] such that more positive scores indicate more positive implicit attitudes toward the left-wing coalition/candidate. Spearman-Brown corrected split-half reliabilities before and after the election were *r*_camp.t1_ = .89, *r*_candidate.t1_ = .72, and *r*_camp.t2_ = .92. Reliabilities were again lower for undecided as compared to decided voters, but not to a large extent (*r*_camp.t1_ = .86 versus.91; *r*_candidate.t1_ = .66 versus.73; *r*_camp.t2_ = .90 versus.92).

Explicit preference of one coalition over the other was assessed with the following question: “After the election, several coalitions are possible. Quite often people talk about a possible ‘red-green’ coalition of SPD and the Greens or a ‘black-yellow’ coalition of CDU/CSU and FDP. Which of the two coalitions do you prefer?” (1 =  I prefer ‘red-green’ very much, 11 =  I prefer ‘black-yellow’ very much). This index was used as the explicit attitude measure in all analyses involving the political camps IAT.

Explicit preference of one candidate over the other was assessed with the following question: “The candidates for chancellor for the next Bundestag election are Angela Merkel and Frank-Walter Steinmeier. Who would you prefer as chancellor?” (1 =  I prefer Steinmeier very much; 11 =  I prefer Merkel very much). Both questions were recoded to match the IAT-coding such that high values indicate a preference for ‘red-green’ and Steinmeier, respectively. This index was used as the explicit attitude measure in all analyses involving the candidates IAT.

To more comprehensively assess the construct explicit attitudes, we constructed an additional explicit measure that was based on separate evaluations of the five major parties of the two political camps. The question read “What do you generally think about the following political parties? What do you think about the …?” The last sentence was repeated for each party. Participants answered an 11-point scale (1 = I have a very negative view of this party; 11 = I have a very positive view of this party). The difference of the weighted means of the evaluations of each party of each political camp served as an indicator of explicit attitudes toward the political camps. The evaluations of the big party in each camp (SPD for the left camp, CDU for the right camp) were weighted with factor two as compared to the smaller parties (Greens for the left camp, CSU and FDP for the right camp). This index was used as an additional, second indicator of explicit attitudes in supplementary analyses involving both the political camps IAT and the candidates IAT (see online supplements, Tables S4, S5, S6, and S7).

Voting intention was assessed with the following question: “Do you already know which party you will vote for in the Bundestag election in September 2009? If yes, which party will that be?” (1 = CDU/CSU, 2 = SPD, 3 = Greens, 4 = FDP, 5 = The Left, 6 = A different party, 7 = I will not vote, 8 = I don’t know yet). Participants who indicated that they did not yet know which party they would vote for were classified as undecided (0). The remaining participants were classified as decided (1).

Voting behavior was assessed with the following questions: “In the Bundestag election you could cast two votes. Your first vote was for a candidate from your electoral district, the second vote for a party. Which party did you give your second vote to?” (1 = CDU/CSU, 2 = SPD, 3 = Greens, 4 = FDP, 5 = The Left, 6 = A different party). For the prediction of voting behavior, we recoded these answers into a dichotomous variable indicating which political camp a participant had voted for. Votes for the CDU/CSU and the FDP were coded as 0 (right-wing camp), votes for the SPD and the Greens were coded as 1 (left-wing camp). All other votes were discarded.

The party The Left is a special case. On the one hand, it belongs to the left political camp and thus should be classified as such. On the other hand, The Left was neither represented in our implicit nor explicit attitude measures. This is why we deemed it more adequate to discard these votes in our analyses. Including participants who had voted for The Left further increases power by adding another 159 participants (102 decideds, 57 undecideds) and leads to very similar results as those reported in Tables 4, 5, 6, and 7.

**Table 4:** Results of the multiple binary logistic regression analyses involving the political camps IAT in Study 2, separately for decided and undecided voters.

Step	Variable	B	*SE*	Wald	*p*	Exp(B)	Nagel-kerke’s R^2^	% CCC
Decided voters (*N* = 408)								
1a	Constant	.008	.145	.003	.955	1.008	.620	84.3
	IAT_camps_	2.422	.225	115.745	<.001	11.270		
1b	Constant	.235	.220	1.144	.285	1.265	.853	93.9
	Explicit_camps_	3.747	.360	108.198	<.001	42.406		
2	Constant	.147	.228	.415	.519	1.158	.860	93.4
	IAT_camps_	.774	.326	5.642	.018	2.169		
	Explicit_camps_	3.266	.386	71.537	<.001	26.210		
Undecided voters (*N* = 202)								
1a	Constant	−.148	.154	.924	.336	.862	.205	70.3
	IAT_camps_	.907	.172	27.773	<.001	2.476		
1b	Constant	−.156	.173	.816	.366	.855	.428	71.8
	Explicit_camps_	2.062	.243	72.164	<.001	7.863		
2	Constant	−.166	.175	.892	.345	.847	.446	72.8
	IAT_camps_	.397	.197	4.057	.044	1.487		
	Explicit_camps_	1.501	.257	34.067	<.001	4.484		

*Note*. B: regression weight B; *SE*: standard error of the regression weight B; Wald: Wald criterion; Exp(B): Odds ratio. Relative amount by which the odds increase (Exp(B) >1.0) or decrease (Exp(B) <1.0) when the value of the predictor is increased by 1 unit; CCC: correctly classified cases; DV: voting behavior (0 =  right political camp, 1 =  left political camp). All continuous variables were z-standardized separately for decided and undecided voters prior to the analyses.

**Table 5:** Results of multiple binary logistic regression analyses involving the political camps IAT in Study 2.

Step	Variable	B	*SE*	Wald	*p*	Exp(B)	Nagel-kerke’s R^2^	% CCC
1	Constant	−.039	.104	.140	.709	.962	.472	80.0
	IAT_camps_	1.747	.139	158.065	<.001	5.736		
2	Constant	−.165	.154	1.150	.284	.848	.505	79.7
	IAT_camps_	.992	.188	27.773	<.001	2.698		
	Decidedness	.194	.211	.840	.359	1.214		
	IAT_camps_* Decidedness	1.334	.287	21.644	<.001	3.797		
3	Constant	−.158	.182	.750	.387	.854	.749	86.7
	IAT_camps_	.382	.219	3.045	.081	1.466		
	Decidedness	.279	.282	.974	.324	1.321		
	IAT_camps_* Decidedness	.456	.358	1.616	.204	1.577		
	Explicit_camps_	2.621	.247	112.827	<.001	13.752		
4	Constant	−.122	.179	.462	.496	.885	.753	86.7
	IAT_camps_	.440	.217	4.115	.043	1.553		
	Decidedness	.343	.294	1.359	.244	1.409		
	IAT_camps_* Decidedness	.239	.386	.385	.535	1.270		
	Explicit_camps_	2.252	.382	34.717	<.001	9.507		
	Explicit_camps_* Decidedness	.751	.511	2.164	.141	2.119		
	IAT_camps_* Explicit_camps_	−.376	.298	1.593	.207	.686		

*Note*. *N* = 610. B: regression weight B; *SE*: standard error of the regression weight B; Wald: Wald criterion; Exp(B): Odds ratio. Relative amount by which the odds increase (Exp(B) >1.0) or decrease (Exp(B) <1.0) when the value of the predictor is increased by 1 unit; CCC: correctly classified cases; DV: voting behavior (0 =  right political camp, 1 =  left political camp). All continuous variables were z-standardized prior to the analyses.

**Table 6:** Results of the multiple binary logistic regression analyses involving the candidates IAT in Study 2, separately for decided and undecided voters.

Step	Variable	B	*SE*	Wald	*p*	Exp(B)	Nagel-kerke’s R^2^	% CCC
Decided voters (*N* = 410)								
1a	Constant	.162	.122	1.776	.183	1.176	.412	79.3
	IAT_candidates_	1.545	.156	98.621	<.001	2.038		
1b	Constant	.366	.153	5.694	.017	1.442	.631	85.6
	Explicit_candidates_	2.325	.204	130.052	<.001	10.227		
2	Constant	.394	.159	6.103	.013	1.483	.658	86.6
	IAT_candidates_	.737	.187	15.607	<.001	2.091		
	Explicit_candidates_	1.975	.215	84.580	<.001	7.206		
Undecided voters (*N* = 210)								
1a	Constant	−.144	.146	.975	.323	.866	.138	65.7
	IAT_candidates_	.712	.159	19.989	<.001	2.038		
1b	Constant	−.157	.150	1.086	.297	.855	.199	64.3
	Explicit_candidates_	.899	.172	27.450	<.001	2.456		
2	Constant	−.164	.154	1.132	.287	.849	.248	67.1
	IAT_candidates_	.498	.168	8.805	.003	1.645		
	Explicit_candidates_	.750	.178	17.790	<.001	2.118		

*Note*. B: regression weight B; *SE*: standard error of the regression weight B; Wald: Wald criterion; Exp(B): Odds ratio. Relative amount by which the odds increase (Exp(B) >1.0) or decrease (Exp(B) <1.0) when the value of the predictor is increased by 1 unit; CCC: correctly classified cases; DV: voting behavior (0 =  right political camp, 1 =  left political camp). All continuous variables were z-standardized separately for decided and undecided voters prior to the analyses.

**Table 7:** Results of multiple binary logistic regression analyses involving the candidates IAT in Study 2.

Step	Variable	B	*SE*	Wald	*p*	Exp(B)	Nagel-kerke’s R^2^	% CCC
1	Constant	.036	.092	.149	.700	1.036	.310	74.0
	IAT_candidates_	1.219	.111	120.220	<.001	3.385		
2	Constant	−.054	.147	.136	.713	.947	.329	74.7
	IAT_candidates_	.739	.165	19.989	<.001	2.093		
	Decidedness	.121	.190	.403	.525	1.128		
	IAT_candidates_* Decidedness	.786	.226	12.134	<.001	2.194		
3	Constant	−.171	.163	1.090	.297	.843	.533	79.4
	IAT_candidates_	.446	.180	6.151	.013	1.562		
	Decidedness	.468	.222	4.429	.035	1.597		
	IAT_candidates_* Decidedness	.358	.248	2.084	.149	1.431		
	Explicit_candidates_	1.503	.147	104.457	<.001	4.496		
4	Constant	−.128	.157	.663	.416	.880	.542	80.2
	IAT_candidates_	.516	.174	8.791	.003	1.675		
	Decidedness	.496	.223	4.933	.026	1.642		
	IAT_candidates_* Decidedness	.217	.258	.703	.402	1.242		
	Explicit_candidates_	.978	.232	17.769	<.001	2.660		
	Explicit_candidates_* Decidedness	.814	.304	7.162	.007	2.258		
	IAT_candidates_* Explicit_candidates_	.017	.164	.011	.917	1.017		

*Note*. *N* = 620. B: regression weight B; *SE*: standard error of the regression weight B; Wald: Wald criterion; Exp(B): Odds ratio. Relative amount by which the odds increase (Exp(B) >1.0) or decrease (Exp(B) <1.0) when the value of the predictor is increased by 1 unit; CCC: correctly classified cases; DV: voting behavior (0 =  right political camp, 1 =  left political camp). All continuous variables were z-standardized prior to the analyses.

### Results and Discussion

#### Preliminary analyses

All continuous variables were z-standardized before running the logistic regression analyses [28]. Participants who completed more than 10% of their trials in less than 300 ms (0.0% in the case of the political camps IAT, 0.32% in the case of the candidates IAT, 0.36% in the case of the post-election political camps IAT) or more than 25% errors in one of the IATs (4.69% in the case of the political camps IAT, 0.33% in the case of the candidates IAT, and 2.4% in the case of the post-election political camps IAT) were excluded from the respective analyses. As in Study 1, the time span between the first measurement and the election was larger for undecided (*M* = 79.00, *SD* = 48.41) as compared to decided participants (*M* = 37.17, *SD* = 35.22; *t*(911) = 14.94, *p*<.001, *d* = 0.99). Controlling for this time span in the joint analyses of decided and undecided voters did not appreciably change the results. This is true for both entering time span as a covariate and as a full factor including all two-way and three-way interactions. These analyses are included in the supplementary online material (–S3).

The political camps IAT and the candidates IAT were substantially correlated, *r* = .60. Both the political camps IATs and the explicit measure showed good stability between the pre- and post-election assessments, *r*_tt-IAT_ = .79 and *r*_tt-EXP_ = .86. Implicit-explicit correspondence was generally high (*r*_camps.t1_ = .67; *r*_camps.t2_ = .72; *r*_candidate.t1_ = .52, all *p*s <.001), and higher among decided as compared to undecided voters (*r*_camps.t1_ = .57 versus.71; *r*_camps.t2_ = .60 versus.77; *r*_candidate.t1_ = .35 versus.59; all *z*-values of comparisons >3.67, all *p*s <.001). This replicates the findings from Study 1 showing that decidedness – perhaps indicating attitude elaboration [24], [29], [30] – is a moderator of implicit-explicit correspondence.

#### Political camps IAT

Again, we started with the investigation of claims 1 and 2: implicit attitudes predict voting behavior better than explicit attitudes for undecided voters and explicit attitudes predict voting behavior better than implicit attitudes for decided voters. Table 4 shows that both implicit and explicit attitudes predicted voting behavior, but to different extents. Implicit attitudes increased Nagelkerke’s *R* ^2^ by 0.7 and 1.8 percentage points for decided and undecided voters, respectively, after accounting for explicit attitudes. The percentage of correctly classified cases increased by 1.0 percentage points for undecided voters and decreased by 0.5 percentage points for decided participants. Explicit attitudes increased Nagelkerke’s *R* ^2^ by 24.0 and 24.1 percentage points for decided and undecided voters, respectively, after accounting for implicit attitudes. The percentage of correctly classified cases increased by 9.1 percentage points for decided and by 2.5 percentage points for undecided voters. Thus, the explicit measure predicted voting behavior better than the implicit measure for both decided and undecided voters, corroborating claim 2 and at odds with claim 1.

Next, we investigated the claims that implicit attitudes predict voting behavior better for undecided than decided voters and that explicit attitudes predict voting behavior better for decided than undecided voters (claims 3 and 4, see Table 5). The IAT predicted voting behavior (Nagelkerke’s *R* ^2^ = .472), correctly classifying 80.0% of the participants’ eventual votes (step 1). In step 2, decidedness moderated the IAT’s influence on voting behavior. As in Study 1, the moderation was in the opposite direction of the results reported by Galdi et al. [7]. The IAT was a better predictor of voting behavior for *decided* as compared to undecided voters, as indicated by the positive regression weight. This interaction remained descriptively in the same direction, but was not statistically significant anymore after explicit attitudes were included in the model (step 3). Adding explicit attitudes increased Nagelkerke’s *R* ^2^ to.749 and the correct classification of votes to 86.7%. Further, implicit attitudes remained a marginally significant, weak predictor of voting. In the final model including all two-way interactions (step 4), the IAT × decidedness interaction remained non-significant as did the explicit × decidedness interaction.

When we entered the second explicit measure based on separate evaluations of the political parties, this measure was highly significant in each case. After including this second explicit measure as an additional predictor in the analyses reported in Tables 4 and 5, all formerly weak, but significant effects of implicit attitudes turned non-significant, indicating that they did not predict voting behavior for either decided or undecided voters after controlling for two indicators of explicit attitudes. These analyses are included in the supplementary online material (Tables S4–S5).

#### Candidates IAT

Similar to the results involving the political camps IAT, both implicit and explicit attitudes predicted voting behavior, albeit to different extents (see Table 6). Controlling for explicit attitudes, implicit attitudes increased Nagelkerke’s *R* ^2^ by 2.7 and 4.9 percentage points for decided and undecided voters. The percentage of correctly classified cases increased by 1.0 versus 2.8 percentage points for decided versus undecided voters. Explicit attitudes increased Nagelkerke’s *R* ^2^ by 24.6 and 11.0 for decided and undecided voters, respectively, after accounting for implicit attitudes. The percentage of correctly classified cases increased by 7.3 and 1.4 percentage points for decided and undecided voters.

In a joint multiple logistic regression analysis of decided and undecided voters, the IAT predicted voting behavior (Nagelkerke’s *R* ^2^ = .310), correctly classifying 74.0% of the participants’ eventual votes (step 1 in Table 7). In step 2, decidedness moderated the IAT’s influence on voting behavior. Again, the IAT was a better predictor of voting behavior for *decided* as compared to undecided voters. This interaction remained descriptively in the same direction, but was not statistically significant anymore after explicit attitudes were included in the model (step 3). Adding explicit attitudes increased Nagelkerke’s *R* ^2^ to.533 and the correct classification of votes to 79.4%. Implicit attitudes remained a significant, but weak predictor of voting. In the final model including all two-way interactions (step 4), the IAT × decidedness interaction remained non-significant and explicit attitudes predicted voting behavior better for decided as compared to undecided voters. In sum, these results largely mirror those from Study 1 and the political camps IAT in Study 2.

As in the case of the political camps IAT, implicit attitudes were a non-significant predictor of voting behavior for either decided or undecided voters in each of the analyses reported in Tables 6 and 7 when we entered the second explicit measure as an additional predictor. These analyses are included in the supplementary online material (Tables S6–S7).

#### Implicit and explicit attitude change as a function of decidedness

**Figure 2:**
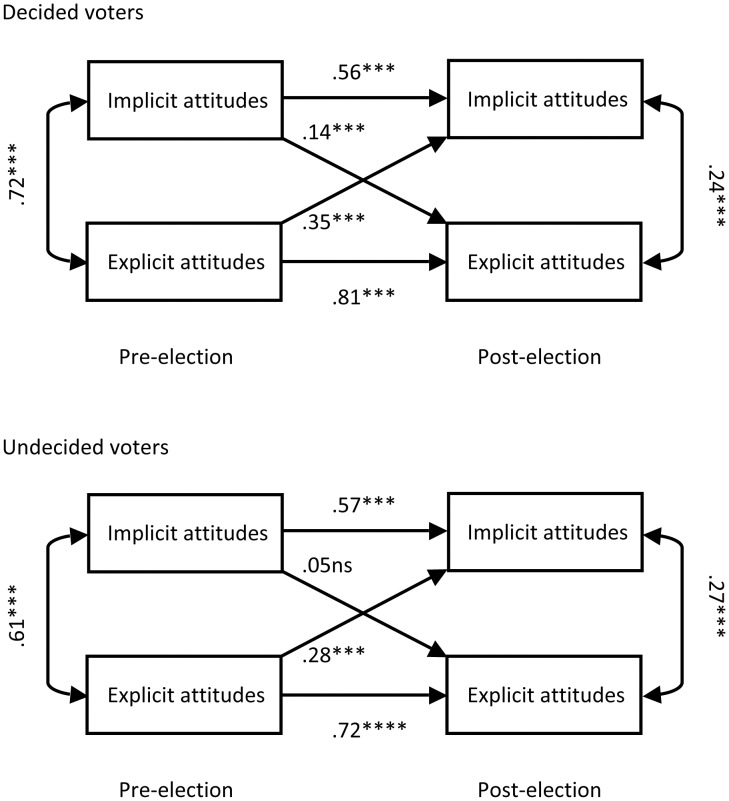
Implicit and explicit attitude change in Study 2. Two-wave-two-variable panel design analysis of implicit and explicit attitude change between the pre- and post-election assessments for decided (*n* = 366) and undecided (*n* = 186) voters in Study 2. Implicit attitudes were indicated by the ‘political camps IAT’. Horizontal arrows indicate stability, diagonal arrows indicate change. Numbers represent standardized beta values of simultaneous multiple regression analyses (***p<.001; ns: not significant).

Similar to Galdi et al. [7], we ran a two-wave-two-variable panel analysis on implicit and explicit attitude change between the two measurement occasions (see Figure 2). In the study by Galdi and colleagues explicit, but not implicit attitudes predicted later explicit attitudes while both explicit and implicit attitudes predicted later implicit attitudes in *decided* individuals. By contrast, implicit, but not explicit attitudes predicted later implicit attitudes while both explicit and implicit attitudes predicted later explicit attitudes in *undecided* individuals.

In this analysis we included all voters who completed the political camps IAT and the explicit measure both pre- and post-election, irrespective of which party they had voted for. Results indicated that pre-election explicit attitudes predicted post-election implicit attitudes for both decided and undecided voters. Pre-election implicit attitudes predicted post-election explicit attitudes, but the relations were weaker and significant only for decided voters. The dynamic of implicit and explicit attitude change as a function of decidedness was apparently very different in the present study than in the studies by Galdi et al. [7], [31].

We conducted a similar analysis of implicit and explicit attitude change between the two pre-election measurements time 1 and time 2 in Study 1. This analysis is similar to the ones in Study 2 and by Galdi et al. [7], [31] with the exception that the second measurement did not take place at the time of election/expression of opinion, respectively. The analysis revealed similar results as the one in Study 2 (Figure 2). The respective figure is included in the supplementary online material (Figure S1).

## General Discussion

The prediction of voting behavior of undecided voters has remained a puzzle for many years. Recently, implicit attitudes have been suggested as an important piece to solve this puzzle. Researchers hypothesized that implicit attitudes indicate which particular candidate or party a voter is leaning to and will ultimately vote for, even if the voter cannot or does not want to commit to a clear voting intention [6]–[8]. If this reasoning held in the context of real political elections, this would represent a major step for the prediction of election outcomes with significant applied implications. We tested this idea in two studies in two different countries with different political systems and using different operationalizations of constructs. Contrary to expectations, explicit attitudes were the stronger predictor of voting behavior for both decided and undecided participants. The increment of implicit attitudes over and above explicit attitudes was significant, but weak in terms of actually increasing the number of correctly classified voters as voting for one or the other candidate or political camp, respectively. In addition, implicit attitudes predicted voting behavior better for *decided* as compared to undecided participants when explicit attitudes were not controlled for. When controlling for explicit attitudes this difference was greatly reduced and there continued to be no evidence for implicit attitudes predicting voting behavior better for undecided than for decided voters. When we put the incremental validity of implicit over explicit attitudes to a more stringent test by controlling for a second indicator of explicit attitudes, each of the formerly significant effects of implicit attitudes turned non-significant. In none of these analyses did implicit attitudes increase the prediction of voting behavior for either decided or undecided voters beyond that accounted for by explicit attitudes.

Finally, Galdi et al. [7] suggested that a specific pattern of implicit and explicit attitude change as a function of decidedness may constitute an important psychological mechanism that may lead to differential predictive validity of explicit and implicit attitudes for decided and undecided voters. A two-wave-two-variable panel analysis in Study 2 suggested that the dynamic between explicit and implicit attitudes was very different than in the study reported by Galdi and colleagues [7]. In their study, implicit attitudes influenced later explicit attitudes of undecided, but not decided voters, and explicit attitudes influenced later implicit attitudes of decided, but not undecided voters. In the present Study 2, explicit attitudes influenced later implicit attitudes for both decided *and* undecided voters, while implicit attitudes had a weaker, but significant effect on later explicit attitudes of *decided* voters, and no significant effect on later explicit attitudes of undecided voters.

### Cognitive Elaboration as an Important Moderator?

The present findings are not simply a failure to replicate previous research. In fact, while Galdi and colleagues [7] predicted *opinions* on a particular topic of local politics, the present studies are the first to test the assumption that implicit, but not explicit attitudes predict *voting behavior* better for undecided than for decided voters in the context of actual political elections. This difference in contexts may be associated with different psychological processes that can explain the respective findings. Thus, the present results do not challenge the pattern of results by Galdi and colleagues. Instead, the present results raise doubts about the presumed implications of the earlier findings for predicting actual voting behavior in the context of major political elections [7], [8].

In the following, we will outline in three steps how we think the degree of cognitive elaboration of the attitudes under investigation may be an important moderator that may contribute to the striking differences in the findings between the present studies and the one by Galdi et al. [7].

#### 1) General political attitudes are more elaborated than attitudes toward particular issues

When voters try to come to a decision for which candidate or party to vote in major political elections they draw on their attitudes toward these candidates, parties, and current political issues. Such political attitudes are often well-elaborated for several reasons: Individuals are frequently confronted with the major political parties and their general positions throughout their lifetime, the candidates are heavily represented in the public media in the run-up to major elections, the current issues are intensively debated. On a basic level, the majority of the electorate can confidently and stably place themselves on a political left-right continuum and this self-placement accounts for a major part of the variance in later voting decisions [22]. Even for undecided voters it is plausible to assume substantial elaboration of political attitudes that may not be as high as for decided voters, but still quite high as compared to other attitude domains for which the attitude objects are not as common in the media and daily conversation across the life span and, in particular, during the run-up to major elections. By contrast, the situation may be quite different for specific political issues of local politics as investigated in the study by Galdi et al. [7]. First, in many cases, such specific issues may pose fairly new questions that individuals did not have the chance to elaborate about for comparable time spans. Second, specific issues may often not receive the same kind of (public and individual) attention and scrutiny, leading to less cognitive elaboration of the attitudes individuals hold about these issues as compared to general political attitudes. Third, while some individuals are closely involved in issues of local politics (and may evolve highly elaborated attitudes), others hardly care about such issues and do not elaborate a great deal on the issues. While there is of course also variance in the degree of elaboration of general political attitudes, the average degree of elaboration is plausibly higher than for issues of local politics.

#### 2) Elaboration is associated with higher implicit-explicit correspondence

Cognitive elaboration of attitudes is associated with a number of related factors such as stability, resistance to persuasion, and attitude strength, all of which foster the correspondence between implicit and explicit attitudes [29], [35]. Indeed, because general political attitudes are rooted in basic psychological needs, they are relatively stable and resistant to long-lasting change [22], [36]. That means that even if cognitive elaboration of political attitudes should be relatively low, there are other factors that may contribute to substantial implicit-explicit correlations in this domain. Consequently, in large-scale studies the domain of political attitudes has revealed implicit-explicit correlations that are among the highest of all investigated domains [19], [29], [37]. Decided voters can be expected to hold even clearer, stronger, more coherent and consistent attitudes than undecided voters. Implicit-explicit consistency should therefore be high overall, but particularly high for decided voters. Indeed, this is the empirical pattern obtained in both of the present studies. Of course, the more two constructs overlap the more difficult it becomes for one construct to explain variance in a criterion over and above the other construct.

#### 3) Higher implicit-explicit correspondence is associated with higher predictive validity of implicit attitudes

A recent meta-analysis revealed that implicit-explicit correspondence was positively associated with predictive validity of implicit attitudes [19]. In fact, both implicit and explicit attitudes in the political domain revealed the highest predictive validities of all investigated domains in this meta-analysis. However, due to the great overlap with explicit attitudes, unique predictive validity of implicit attitudes in the political domain was greatly reduced to the average of all domains once explicit attitudes were controlled for. By contrast, even controlling for implicit attitudes, explicit attitudes in the political domain still showed the highest predictive validity of all domains. The present data correspond to these meta-analytic findings in that implicit attitudes predicted voting behavior better for decided than for undecided voters, but this difference was greatly reduced once explicit attitudes were controlled for.

Taken together, this analysis of the literature illustrates how dramatically reversed these meta-analytic interrelations would have to be for implicit attitudes to predict (a) voting behavior better than explicit attitudes for undecided voters, and/or (b) better for undecided as compared to decided voters. No analysis showed evidence for such a dramatic turnaround. Although the outlined reasoning is in line with current theorizing in implicit social cognition and the current data, due to the correlational evidence, no strong conclusions can be drawn until experimental research has provided more comprehensive tests of these ideas.

How can the present data be reconciled with those obtained by Galdi and colleagues [7]? Our theoretical analysis suggests that in contexts of high implicit-explicit correspondence such as well-elaborated political attitudes, it will be difficult for implicit attitudes to explain substantial variance over and above explicit attitudes. This is indeed what the present data show. Although implicit attitudes were a significant predictor over and above explicit attitudes and various interactions with decidedness in several analyses, their contribution to the overall quality of the predictions was weak. By contrast, in contexts of relatively low implicit-explicit correspondence there is a greater chance for implicit attitudes to explain variance in a behavior over and above explicit attitudes. In such contexts, it seems plausible that undecided voters draw on their implicit attitudes to reach a decision, as predicted by Galdi et al. [7]. In the present studies, this view is partly corroborated by slightly higher incremental validity of implicit over explicit attitudes for undecided as compared to decided voters in several analyses. However, even for undecided voters implicit-explicit consistency was very substantial, precluding appreciable incremental validity of implicit over explicit attitudes. By contrast, in the study by Galdi and colleagues, implicit-explicit correspondence was quite low (*r*s <.20) and non-significant for both decided and undecided voters, allowing for considerable incremental validity of implicit over explicit attitudes for undecided voters. Implicit-explicit correspondence can be low due to a variety of reasons, including weak and weakly elaborated attitudes, strong self-presentational demands, particularly controversial contexts that make a dissociation of implicit and explicit attitudes more likely [29], . Such implicit-explicit dissociations may contribute to feeling undecided about an issue [25], possibly because individuals experience the incoherence of implicit and explicit attitudes as an inner conflict of competing tendencies that impedes the smooth expression of behavioral intentions and actual behavior.

This view is also in line with current dual-process models of information processing such as the reflective-impulsive model (RIM) [38]. Consistent with our reasoning, the RIM assumes that a repeated cognitive preoccupation with issues will slowly align reflective and impulsive structures, increasing implicit-explicit consistency in the respective domains. High implicit-explicit consistency, that is, a compatibility of impulsive (implicit) and reflective (explicit) processes will facilitate, and a dissociation will effectively inhibit the execution of behavior. Given dissociation, either implicit or explicit processes will predominantly guide behavior, depending on several boundary conditions. Deliberate, controlled behavior should be greatly influenced by reflective, explicit processes. By contrast, more impulsive behavior due to a lack of an individual’s motivation and/or ability to control a particular behavior should be greatly influenced by impulsive, implicit processes. A large body of literature is consistent with these assumptions [20], [21]. From this perspective, the findings by Galdi and colleagues [7] remain particularly intriguing. Implicit and explicit attitudes were largely dissociated, indicating a potential for differential predictive validity. However, the behavior (stating one’s opinion on a political question) was largely deliberate and controlled. Nevertheless, undecided participants’ opinions were predicted by their implicit attitudes, assessed one week before.

### Alternative Explanations and Limitations

As noted earlier, the present studies were not intended to provide a direct replication of the work by Galdi et al. [7], but a test of claims that were made based on this work. Nevertheless, one could argue that one reason why in the present studies explicit attitudes outperformed implicit attitudes in predicting self-reported voting behavior was that the measures used in the present studies were substantially more “affective” and thus more closely related to implicit attitudes [39], [40]. From this perspective, our presumably affectively-toned explicit measures were the “better implicit measures” by capturing affective gut responses even more efficiently than the IATs. By contrast, the explicit measure in the study by Galdi et al. was more cognitively-toned and asked for assumed consequences of the enlargement of the military base. The bottom line of this argument would be that in fact there are no inconsistencies between the current studies and the earlier one by Galdi and colleagues.

For several reasons, we do not believe that this reasoning can explain the apparent differences in results between the studies. We acknowledge the difference in the explicit measures employed. The feeling thermometer in Study 1 is indeed an affectively-based explicit measure. However, neither the preference measure used as the primary explicit measure, nor the second explicit measure based on party-evaluations in Study is particularly affectively-based. The argument therefore applies much less to Study 2. Even more important, however, is that irrespective of any explicit measure implicit attitudes predicted self-reported voting behavior better for *decided* as compared to undecided participants, which is in stark contrast to the theoretical reasoning and empirical findings brought forward by Galdi and colleagues [7], [8]. Above, we outlined one reason why this may be the case, and we remain open to alternative explanations that are independent from explanations based on high implicit-explicit correlations in the political domain.

Another factor that may have contributed to differences in results between the study by Galdi et al. [7] and the present studies is the difference in sample sizes. While the sample sizes in Galdi et al.’s study were fairly small especially for undecided voters (*N*_undecided_  = 33; *N*_decided_  = 96), sample sizes were considerably larger in the present studies (ranging from 202 to 303 for undecided voters and between 408 and 3291 for decided voters). Small sample sizes can lead to instable estimates in regression analyses [41]. Reassuringly, the pattern of implicit and explicit attitude change reported by Galdi et al. [7] has recently been replicated and shown to be mediated by differences in selective exposure to different kinds of persuasive information by decided and undecided individuals in a somewhat larger sample (*N*_undecided_  = 53; *N*_decided_  = 60) [31]. The replication of the prediction of opinion as a function of the decidedness was not in focus in the latter study.

One limitation of Study 2 concerns the item used to assess decidedness. This item asked whether or not participants had already decided which party they would vote for or if they were still undecided. This item allowed for some ambiguity insofar as a person may not have had decided which party to vote for even though s/he may have had decided which coalition – i.e., the undecided component could be between CDU/CSU and FDP. This means that there could be some participants in the ‘undecided’ group that were already decided on the coalition and candidate dimensions, but not the party dimension. For several reasons, we think that this ambiguity does not threaten the interpretation of Study 2’s results. First, the decidedness item in Study 1 was unambiguous and results were consistent across both studies. Second, in both studies explicit attitudes were the better predictor than parallel implicit attitudes for undecided voters’ voting behavior. From a theoretical perspective, it does not seem plausible to assume that this would generally be true for participants who are undecided which party to vote for, but dramatically reversed for participants who know which coalition to vote for, but not yet which party within the coalition. Third, previous research in the context of the German party system showed that within-camp correlations of implicit attitudes toward the major political parties are positive and between-camp correlations are negative. Correlations between implicit attitudes toward the CDU and the FDP (the right-wing camp) on the one side and the SPD and the Greens (the left-wing camp) one the other side, were consistently the highest of all correlations between the major parties [18]. This suggests that even if a voter was decided for the political camp, but undecided about the particular party within the camp, implicit attitudes toward the parties within the camp will be reliably positively related and therefore predict voting behavior in a similar direction. Taken together, it does not seem likely that the slight ambiguity of the decidedness item in Study 2 appreciably influenced the results of the study.

### Conclusion

Implicit attitudes have been argued to predict the voting behavior of undecided voters better than explicit attitudes, thereby solving a long-standing problem in polling research. The present studies were the first to test this assumption in the context of real political elections. Explicit attitudes outperformed implicit attitudes in predicting the voting behavior of both decided and undecided voters and implicit attitudes predicted voting behavior better for decided, not undecided voters. While implicit attitudes may yet contribute to political prediction in other ways, these results suggest that there remains a puzzle of reliably predicting the vote of undecided voters.

## Supporting Information

Figure S1 **Implicit and explicit attitude change in Study 1.** Two-wave-two-variable panel design analysis of implicit and explicit attitude change between time 1 and time 2 for decided (*n* = 920) and undecided (*n* = 86) voters in Study 1. Horizontal arrows indicate stability, diagonal arrows indicate change. Numbers represent standardized beta values of simultaneous multiple regression analyses (***p<.001; ns: not significant).
(TIF)

Table S1 **Results of multiple binary logistic regression analyses in Study 1 including both decided and undecided voters, controlling for the time span between the first measurement and the election.** This table corresponds to Table 3 in the main manuscript.
(DOC)

Table S2 **Results of multiple binary logistic regression analyses involving the political camps IAT in Study 2, controlling for the time span between the first measurement and the election.** This table corresponds to Table 5 in the main manuscript.
(DOC)

Table S3 **Results of multiple binary logistic regression analyses involving the candidates IAT in Study 2, controlling for the time span between the first measurement and the election.** This table corresponds to Table 7 in the main manuscript.
(DOC)

Table S4 **Results of the multiple binary logistic regression analyses involving the political camps IAT in Study 2, separately for decided and undecided voters and including a second indicator of explicit attitudes (Explicit_party-based_, see main manuscript for details).** This table corresponds to Table 4 in the main manuscript.
(PDF)

Table S5 **Results of multiple binary logistic regression analyses involving the political camps IAT in Study 2, including a second indicator of explicit attitudes (Explicit_party-based_, see main manuscript for details).** This table corresponds to Table 5 in the main manuscript.
(PDF)

Table S6 **Results of the multiple binary logistic regression analyses involving the candidates IAT in Study 2, separately for decided and undecided voters and including a second indicator of explicit attitudes (Explicit_party-based_, see main manuscript for details).** This table corresponds to Table 6 in the main manuscript.
(PDF)

Table S7 **Results of multiple binary logistic regression analyses involving the candidates IAT in Study 2, including a second indicator of explicit attitudes (Explicit_party-based_, see main manuscript for details).** This table corresponds to Table 7 in the main manuscript.
(PDF)
