# Supplementary figures and images for: Do Implicit Attitudes Predict Actual Voting Behavior Particularly for Undecided Voters?
Source: PLoS One. 2012 Aug 29;7(8):e44130. doi: 10.1371/journal.pone.0044130 (PMC3430672; doi:10.1371/journal.pone.0044130)

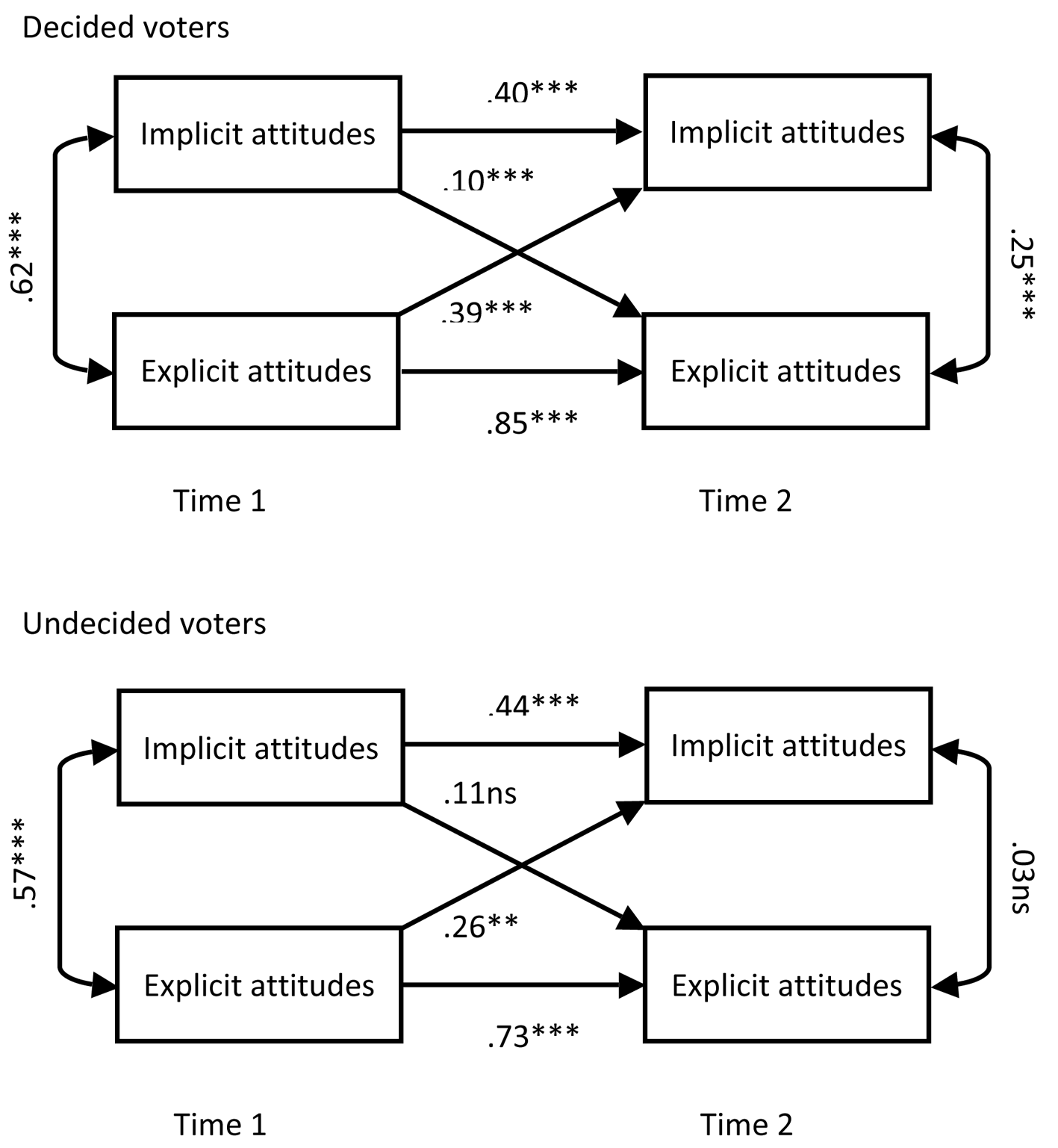

Supplement: Figure S1 — Implicit and explicit attitude change in Study 1. Two-wave-two-variable panel design analysis of implicit and explicit attitude change between time 1 and time 2 for decided (n = 920) and undecided (n = 86) voters in Study 1. Horizontal arrows indicate stability, diagonal arrows indicate change. Numbers represent standardized beta values of simultaneous multiple regression analyses (***p<.001; ns: not significant). (TIF) [file pone.0044130.s001.tif]
