# Supplementary material for: Do Implicit Attitudes Predict Actual Voting Behavior Particularly for Undecided Voters?
Source: PLoS One. 2012 Aug 29;7(8):e44130. doi: 10.1371/journal.pone.0044130 (PMC3430672; doi:10.1371/journal.pone.0044130)
Supplement: Table S1 — Results of multiple binary logistic regression analyses in Study 1 including both decided and undecided voters, controlling for the time span between the first measurement and the election. This table corresponds to Table 3 in the main manuscript. (DOC) [file pone.0044130.s002.doc]

Table S1. Results of multiple binary logistic regression analyses in Study 1 including both decided and undecided voters, controlling for the time span between the first measurement and the election. This table corresponds to Table 3 in the main manuscript.

| Step | Variable | B | *SE* | Wald | *p* | Exp(B) | Nagel-kerke’s R^2^ | % CCC |
| --- | --- | --- | --- | --- | --- | --- | --- | --- |
| 0 | Constant | 1.671 | .046 | 1336.638 | < .001 | 5.318 | < .001 | 84.2 |
|  | Time | -.033 | .046 | .511 | .475 | .968 |  |  |
| 1 | Constant | 2.889 | .096 | 914.464 | < .001 | 17.981 | .538 | 89.4 |
|  | Time | .010 | .061 | .025 | .874 | 1.010 |  |  |
|  | IAT | 2.211 | .086 | 663.863 | < .001 | 9.126 |  |  |
| 2 | Constant | 1.660 | .206 | 65.075 | < .001 | 5.259 | .549 | 89.5 |
|  | Time | .019 | .061 | .094 | .759 | 1.019 |  |  |
|  | IAT | 1.177 | .183 | 41.201 | < .001 | 3.246 |  |  |
|  | Decidedness | 1.438 | .234 | 37.889 | < .001 | 4.212 |  |  |
|  | IAT*Decidedness | 1.215 | .209 | 33.944 | < .001 | 3.371 |  |  |
| 3 | Constant | 4.514 | .310 | 211.412 | < .001 | 91.256 | .852 | 97.1 |
|  | Time | .131 | .102 | 1.628 | .202 | 1.140 |  |  |
|  | IAT | .538 | .235 | 5.250 | .022 | 1.712 |  |  |
|  | Decidedness | .149 | .306 | .239 | .625 | 1.161 |  |  |
|  | IAT* Decidedness | .260 | .283 | .845 | .358 | 1.297 |  |  |
|  | Explicit | 3.815 | .202 | 355.295 | < .001 | 45.389 |  |  |
| 4 | Constant | 3.318 | .405 | 66.962 | < .001 | 27.595 | .856 | 97.1 |
|  | Time | .126 | .105 | 1.462 | .227 | 1.135 |  |  |
|  | IAT | -.070 | .299 | .055 | .814 | .932 |  |  |
|  | Decidedness | 1.369 | .468 | 8.566 | .003 | 3.931 |  |  |
|  | IAT* Decidedness | .171 | .286 | .356 | .551 | 1.186 |  |  |
|  | Explicit | 2.338 | .428 | 29.794 | < .001 | 10.361 |  |  |
|  | Explicit* Decidedness | 1.360 | .478 | 8.096 | .004 | 3.898 |  |  |
|  | IAT* Explicit | -.819 | .244 | 11.287 | .001 | .441 |  |  |
| 5 | Constant | 3.330 | .409 | 66.333 | < .001 | 27.927 | .856 | .971 |
|  | Time | .149 | .256 | .342 | .559 | 1.161 |  |  |
|  | IAT | -.093 | .300 | .096 | .757 | .911 |  |  |
|  | Decidedness | 1.375 | .471 | 8.515 | .004 | 3.953 |  |  |
|  | IAT* Decidedness | .169 | .285 | .352 | .553 | 1.184 |  |  |
|  | Explicit | 2.378 | .432 | 30.347 | < .001 | 10.788 |  |  |
|  | Explicit* Decidedness | 1.354 | .479 | 7.991 | .005 | 3.874 |  |  |
|  | IAT* Explicit | -.808 | .245 | 10.915 | .001 | .446 |  |  |
|  | IAT * Time | .157 | .139 | 1.272 | .259 | 1.170 |  |  |
|  | Decidedness * Time | -.049 | .220 | .049 | .825 | .952 |  |  |
|  | Explicit * Time | -.113 | .211 | .289 | .591 | .893 |  |  |
| 6 | Constant | 3.623 | .520 | 48.599 | < .001 | 37.460 | .858 | 97.1 |
|  | Time | .419 | .449 | .868 | .351 | 1.520 |  |  |
|  | IAT | .359 | .512 | .492 | .483 | 1.432 |  |  |
|  | Decidedness | 1.162 | .575 | 4.087 | .043 | 3.198 |  |  |
|  | IAT* Decidedness | -.398 | .578 | .474 | .491 | .672 |  |  |
|  | Explicit | 2.768 | .579 | 22.890 | < .001 | 15.924 |  |  |
|  | Explicit* Decidedness | .971 | .636 | 2.331 | .127 | 2.641 |  |  |
|  | IAT* Explicit | -.269 | .530 | .258 | .612 | .764 |  |  |
|  | IAT * Time | .470 | .305 | 2.375 | .123 | 1.600 |  |  |
|  | Decidedness * Time | -.356 | .510 | .488 | .485 | .701 |  |  |
|  | Explicit * Time | -.040 | .295 | .018 | .893 | .961 |  |  |
|  | Explicit* Decidedness*Time | -.279 | .484 | .332 | .564 | .756 |  |  |
|  | IAT*Explicit*Time | .390 | .222 | 3.088 | .079 | 1.477 |  |  |
|  | IAT*Decidedness* Time | -.737 | .595 | 1.537 | .215 | .478 |  |  |
| *Note*. *N* = 3594. B: regression weight B; *SE*: standard error of the regression weight B; Wald: Wald criterion; Exp(B): Odds ratio. Relative amount by which the odds increase (Exp(B) > 1.0) or decrease (Exp(B) < 1.0) when the value of the predictor is increased by 1 unit; CCC: correctly classified cases; time: time span between the first measurement and the election; DV: voting behavior (0 = McCain, 1 = Obama). The IAT, explicit measure and decidedness information used in this analysis was obtained at time 1. All continuous variables were z-standardized prior to the analyses. | | | | | | | | |
