# Supplementary material for: Do Implicit Attitudes Predict Actual Voting Behavior Particularly for Undecided Voters?
Source: PLoS One. 2012 Aug 29;7(8):e44130. doi: 10.1371/journal.pone.0044130 (PMC3430672; doi:10.1371/journal.pone.0044130)
Supplement: Table S3 — Results of multiple binary logistic regression analyses involving the candidates IAT in Study 2, controlling for the time span between the first measurement and the election. This table corresponds to Table 7 in the main manuscript. (DOC) [file pone.0044130.s004.doc]

Table S3. Results of multiple binary logistic regression analyses involving the candidates IAT in Study 2, controlling for the time span between the first measurement and the election. This table corresponds to Table 7 in the main manuscript.

| Step | Variable | B | *SE* | Wald | *p* | Exp(B) | Nagel-kerke’s R^2^ | % CCC |
| --- | --- | --- | --- | --- | --- | --- | --- | --- |
| 0 | Constant | .013 | .080 | .026 | .872 | 1.013 | < .001 | 54.2 |
|  | Time | .026 | .080 | .108 | .742 | 1.027 |  |  |
| 1 | Constant | .034 | .092 | .136 | .713 | 1.035 | .312 | 74.0 |
|  | Time | .093 | .092 | 1.024 | .312 | 1.098 |  |  |
|  | IAT_candidates_ | 1.225 | .111 | 120.829 | < .001 | 3.405 |  |  |
| 2 | Constant | -.141 | .163 | .750 | .387 | .868 | .332 | 74.7 |
|  | Time | .131 | .103 | 1.596 | .207 | 1.140 |  |  |
|  | IAT_candidates_ | .745 | .166 | 20.156 | < .001 | 2.106 |  |  |
|  | Decidedness | .250 | .217 | 1.331 | .249 | 1.284 |  |  |
|  | IAT_candidates_* Decidedness | .781 | .226 | 11.962 | .001 | 2.184 |  |  |
| 3 | Constant | -.261 | .182 | 2.054 | .152 | .770 | .535 | 80.5 |
|  | Time | .133 | .117 | 1.296 | .255 | 1.142 |  |  |
|  | IAT_candidates_ | .460 | .181 | 6.450 | .011 | 1.584 |  |  |
|  | Decidedness | .596 | .250 | 5.682 | .017 | 1.815 |  |  |
|  | IAT_candidates_* Decidedness | .343 | .249 | 1.898 | .168 | 1.410 |  |  |
|  | Explicit_candidates_ | 1.502 | .147 | 104.254 | < .001 | 4.492 |  |  |
| 4 | Constant | -.213 | .176 | 1.475 | .225 | .808 | .544 | 81.5 |
|  | Time | .126 | .115 | 1.212 | .271 | 1.134 |  |  |
|  | IAT_candidates_ | .527 | .175 | 9.065 | .003 | 1.693 |  |  |
|  | Decidedness | .614 | .249 | 6.106 | .013 | 1.848 |  |  |
|  | IAT_candidates_* Decidedness | .206 | .259 | .632 | .427 | 1.229 |  |  |
|  | Explicit_candidates_ | .980 | .233 | 17.738 | < .001 | 2.664 |  |  |
|  | Explicit_candidates_* Decidedness | .809 | .305 | 7.046 | .008 | 2.245 |  |  |
|  | IAT_candidates_* Explicit_candidates_ | .024 | .165 | .021 | .886 | 1.024 |  |  |
| 5 | Constant | -.193 | .188 | 1.057 | .304 | .824 | .546 | 80.6 |
|  | Time | .071 | .145 | .237 | .626 | 1.073 |  |  |
|  | IAT_candidates_ | .628 | .196 | 10.264 | .001 | 1.874 |  |  |
|  | Decidedness | .604 | .251 | 5.782 | .016 | 1.829 |  |  |
|  | IAT_candidates_* Decidedness | .071 | .281 | .063 | .801 | 1.073 |  |  |
|  | Explicit_candidates_ | 1.002 | .254 | 15.615 | < .001 | 2.724 |  |  |
|  | Explicit_candidates_* Decidedness | .769 | .339 | 5.152 | .023 | 2.158 |  |  |
|  | IAT_candidates_* Explicit_candidates_ | .040 | .166 | .058 | .809 | 1.041 |  |  |
|  | Explicit_candidates_* Time | -.022 | .164 | .017 | .895 | .979 |  |  |
|  | Decidedness*Time | .106 | .250 | .180 | .672 | 1.112 |  |  |
|  | IAT_candidates_*Time | -.167 | .133 | 1.570 | .210 | .846 |  |  |
| 6 | Constant | -.231 | .188 | 1.506 | .220 | .794 | .554 | 81.1 |
|  | Time | .063 | .150 | .175 | .676 | 1.065 |  |  |
|  | IAT_candidates_ | .556 | .205 | 7.339 | .007 | 1.744 |  |  |
|  | Decidedness | .698 | .258 | 7.305 | .007 | 2.010 |  |  |
|  | IAT_candidates_* Decidedness | .049 | .282 | .030 | .861 | 1.050 |  |  |
|  | Explicit_candidates_ | 1.042 | .266 | 15.364 | < .001 | 2.835 |  |  |
|  | Explicit_candidates_* Decidedness | .776 | .341 | 5.171 | .023 | 2.173 |  |  |
|  | IAT_candidates_* Explicit_candidates_ | .287 | .266 | 1.160 | .281 | 1.332 |  |  |
|  | Explicit_candidates_* Time | .016 | .217 | .005 | .942 | 1.016 |  |  |
|  | Decidedness*Time | .092 | .256 | .128 | .721 | 1.096 |  |  |
|  | IAT_candidates_*Time | -.074 | .169 | .194 | .659 | .928 |  |  |
|  | Explicit_candidates_* Decidedness*Time | -.028 | .350 | .006 | .936 | .972 |  |  |
|  | IAT_candidates_* Explicit_candidates_* Time | .200 | .191 | 1.104 | .293 | 1.222 |  |  |
|  | IAT_candidates_* Decidedness*Time | -.289 | .276 | 1.094 | .296 | .749 |  |  |
|  | IAT_candidates_* Explicit_candidates_* Decidedness | -.467 | .360 | 1.689 | .194 | .627 |  |  |
| *Note*. *N* = 620. B: regression weight B; *SE*: standard error of the regression weight B; Wald: Wald criterion; Exp(B): Odds ratio. Relative amount by which the odds increase (Exp(B) > 1.0) or decrease (Exp(B) < 1.0) when the value of the predictor is increased by 1 unit; CCC: correctly classified cases; time: time span between the first measurement and the election; DV: voting behavior (0 = right political camp, 1 = left political camp). All continuous variables were z-standardized prior to the analyses. | | | | | | | | |
