# Supplementary material for: Do Implicit Attitudes Predict Actual Voting Behavior Particularly for Undecided Voters?
Source: PLoS One. 2012 Aug 29;7(8):e44130. doi: 10.1371/journal.pone.0044130 (PMC3430672; doi:10.1371/journal.pone.0044130)
Supplement: Table S4 — Results of the multiple binary logistic regression analyses involving the political camps IAT in Study 2, separately for decided and undecided voters and including a second indicator of explicit attitudes (Explicitparty-based, see main manuscript for details). This table corresponds to Table 4 in the main manuscript. (PDF) [file pone.0044130.s005.pdf]

Table S4. Results of the multiple binary logistic regression analyses involving the political camps IAT in Study 2, separately for decided and undecided voters and including a second indicator of explicit attitudes ($Explicit_{party\text{-}based}$, see main manuscript for details). This table corresponds to Table 4 in the main manuscript.

Step	Variable	B	*SE*	Wald	*p*	Exp(B)	Nagel-kerke's R^2	% CCC
Decided voters (*N* = 408)								
1a	Constant	.008	.145	.003	.955	1.008	.620	84.3
	IAT_{camps}	2.422	.225	115.745	< .001	11.270		
1b	Constant	.169	.234	.517	.472	1.184	.877	94.6
	$Explicit_{camps}$	2.197	.424	26.882	< .001	9.002		
	$Explicit_{party\text{-}based}$	2.706	.617	19.218	< .001	14.968		
2	Constant	.124	.239	.271	.603	1.132	.879	94.6
	IAT_{camps}	.485	.338	2.069	.150	1.625		
	$Explicit_{camps}$	2.024	.436	21.529	< .001	7.568		
	$Explicit_{party\text{-}based}$	2.512	.632	15.798	< .001	12.332		
Undecided voters (*N* = 202)								
1a	Constant	-.148	.154	.924	.336	.862	.205	70.3
	IAT_{camps}	.907	.172	27.773	< .001	2.476		
1b	Constant	-.235	.190	1.520	.218	.791	.557	80.2
	$Explicit_{camps}$	.498	.325	2.351	.125	1.645		
	$Explicit_{party\text{-}based}$	2.167	.457	22.532	< .001	8.733		
2	Constant	-.237	.190	1.548	.213	.789	.558	81.2
	IAT_{camps}	-.082	.235	.121	.728	.921		

$\text{Explicit}_{\text{camps}}$	.496	.324	2.342	.126	1.643
$\text{Explicit}_{\text{party-based}}$	2.235	.500	20.011	< .001	9.345

Note. B: regression weight B; *SE*: standard error of the regression weight B; Wald: Wald criterion; Exp(B): Odds ratio. Relative amount by which the odds increase (Exp(B) > 1.0) or decrease (Exp(B) < 1.0) when the value of the predictor is increased by 1 unit; CCC: correctly classified cases; DV: voting behavior (0 = right political camp, 1 = left political camp). All continuous variables were z-standardized separately for decided and undecided voters prior to the analyses.
