# Supplementary material for: Do Implicit Attitudes Predict Actual Voting Behavior Particularly for Undecided Voters?
Source: PLoS One. 2012 Aug 29;7(8):e44130. doi: 10.1371/journal.pone.0044130 (PMC3430672; doi:10.1371/journal.pone.0044130)
Supplement: Table S5 — Results of multiple binary logistic regression analyses involving the political camps IAT in Study 2, including a second indicator of explicit attitudes (Explicitparty-based, see main manuscript for details). This table corresponds to Table 5 in the main manuscript. (PDF) [file pone.0044130.s006.pdf]

Table S5. Results of multiple binary logistic regression analyses involving the political camps IAT in Study 2, including a second indicator of explicit attitudes ($Explicit_{party\text{-}based}$, see main manuscript for details). This table corresponds to Table 5 in the main manuscript.

Step	Variable	B	*SE*	Wald	*p*	Exp(B)	Nagel-kerke's R^2	% CCC
1	Constant	-.039	.104	.140	.709	.962	.472	80.0
	IAT_{camps}	1.747	.139	158.065	< .001	5.736		
2	Constant	-.165	.154	1.150	.284	.848	.505	79.7
	IAT_{camps}	.992	.188	27.773	< .001	2.698		
	Decidedness	.194	.211	.840	.359	1.214		
	IAT_{camps}*Decidedness	1.334	.287	21.644	< .001	3.797		
3	Constant	-.260	.190	1.873	.171	.771	.789	89.5
	IAT_{camps}	-.010	.239	.002	.967	.990		
	Decidedness	.370	.301	1.510	.219	1.447		
	IAT_{camps}* Decidedness	.509	.381	1.785	.182	1.664		
	$Explicit_{camps}$	1.408	.295	22.760	< .001	14.088		
	$Explicit_{party\text{-}based}$	2.673	.453	34.811	< .001	14.488		
4	Constant	-.220	.188	1.364	.243	.803	.792	90.0
	IAT_{camps}	.037	.238	0.25	.875	1.038		
	Decidedness	.450	.314	2.053	.152	1.568		
	IAT_{camps}* Decidedness	.269	.406	.439	.508	1.309		
	$Explicit_{camps}$	1.072	.436	6.039	.014	2.923		

$Explicit_{party\text{-}based}$	2.792	.472	34.934	< .001	16.313
$Explicit_{camps}$* Decidedness	.709	.552	1.650	.199	2.032
IAT_{camps}* $Explicit_{camps}$	-.508	.325	2.450	.118	.602

Note. *N* = 610. B: regression weight B; *SE*: standard error of the regression weight B; Wald: Wald criterion; Exp(B): Odds ratio. Relative amount by which the odds increase (Exp(B) > 1.0) or decrease (Exp(B) < 1.0) when the value of the predictor is increased by 1 unit; CCC: correctly classified cases; DV: voting behavior (0 = right political camp, 1 = left political camp). All continuous variables were z-standardized prior to the analyses.
