# Supplementary material for: Do Implicit Attitudes Predict Actual Voting Behavior Particularly for Undecided Voters?
Source: PLoS One. 2012 Aug 29;7(8):e44130. doi: 10.1371/journal.pone.0044130 (PMC3430672; doi:10.1371/journal.pone.0044130)
Supplement: Table S6 — Results of the multiple binary logistic regression analyses involving the candidates IAT in Study 2, separately for decided and undecided voters and including a second indicator of explicit attitudes (Explicitparty-based, see main manuscript for details). This table corresponds to Table 6 in the main manuscript. (PDF) [file pone.0044130.s007.pdf]

Table S6. Results of the multiple binary logistic regression analyses involving the candidates IAT in Study 2, separately for decided and undecided voters and including a second indicator of explicit attitudes ($Explicit_{party\text{-}based}$, see main manuscript for details). This table corresponds to Table 6 in the main manuscript.

Step	Variable	B	*SE*	Wald	*p*	Exp(B)	Nagel-kerke's R^2	% CCC
Decided voters (*N* = 410)								
1a	Constant	.162	.122	1.776	.183	1.176	.412	79.3
	$IAT_{candidates}$	1.545	.156	98.621	< .001	2.038		
1b	Constant	.183	.217	.714	.398	1.201	.849	92.9
	$Explicit_{candidates}$	.910	.297	9.378	.002	2.484		
	$Explicit_{party\text{-}based}$	4.203	.561	56.189	< .001	66.918		
2	Constant	.200	.220	.834	.361	1.222	.851	93.7
	$IAT_{candidates}$	.298	.250	1.421	.233	1.347		
	$Explicit_{candidates}$	.830	.304	7.465	.006	2.294		
	$Explicit_{party\text{-}based}$	4.065	.557	53.233	< .001	58.258		
Undecided voters (*N* = 210)								
1a	Constant	-.144	.146	.975	.323	.866	.138	65.7
	$IAT_{candidates}$	.712	.159	19.989	< .001	2.038		
1b	Constant	-.263	.182	2.077	.150	.769	.526	81.4
	$Explicit_{candidates}$	.147	.218	.455	.500	1.158		
	$Explicit_{party\text{-}based}$	2.342	.383	37.458	< .001	10.405		
2	Constant	-.254	.183	1.923	.165	.776	.527	81.0
	$IAT_{candidates}$	.114	.205	.306	.580	1.120		

$Explicit_{candidates}$	.120	.224	.287	.592	1.127
$Explicit_{party\text{-}based}$	2.288	.392	34.147	< .001	9.856

Note. B: regression weight B; *SE*: standard error of the regression weight B; Wald: Wald criterion; Exp(B): Odds ratio. Relative amount by which the odds increase (Exp(B) > 1.0) or decrease (Exp(B) < 1.0) when the value of the predictor is increased by 1 unit; CCC: correctly classified cases; DV: voting behavior (0 = right political camp, 1 = left political camp). All continuous variables were z-standardized separately for decided and undecided voters prior to the analyses.
