# Supplementary material for: Do Implicit Attitudes Predict Actual Voting Behavior Particularly for Undecided Voters?
Source: PLoS One. 2012 Aug 29;7(8):e44130. doi: 10.1371/journal.pone.0044130 (PMC3430672; doi:10.1371/journal.pone.0044130)
Supplement: Table S7 — Results of multiple binary logistic regression analyses involving the candidates IAT in Study 2, including a second indicator of explicit attitudes (Explicitparty-based, see main manuscript for details). This table corresponds to Table 7 in the main manuscript. (PDF) [file pone.0044130.s008.pdf]

Table S7. Results of multiple binary logistic regression analyses involving the candidates IAT in Study 2, including a second indicator of explicit attitudes ($Explicit_{party\text{-}based}$, see main manuscript for details). This table corresponds to Table 7 in the main manuscript.

Step	Variable	B	*SE*	Wald	*p*	Exp(B)	Nagelkerke's R^2	% CCC
1	Constant	.036	.092	.149	.700	1.036	.310	74.0
	$IAT_{candidates}$	1.219	.111	120.220	< .001	3.385		
2	Constant	-.054	.147	.136	.713	.947	.329	74.7
	$IAT_{candidates}$	.739	.165	19.989	< .001	2.093		
	Decidedness	.121	.190	.403	.525	1.128		
	$IAT_{candidates}$* Decidedness	.786	.226	12.134	< .001	2.194		
3	Constant	-.321	.189	2.899	.089	.725	.758	89.4
	$IAT_{candidates}$	.058	.207	.079	.778	1.060		
	Decidedness	.481	.284	2.874	.090	1.618		
	$IAT_{candidates}$* Decidedness	.294	.309	.908	.341	1.342		
	$Explicit_{candidates}$	.479	.201	5.673	.017	1.615		
	$Explicit_{party\text{-}based}$	3.612	.387	87.166	< .001	37.040		
4	Constant	-.301	.191	2.488	.115	.740	.760	89.5
	$IAT_{candidates}$	.124	.210	.348	.556	1.132		
	Decidedness	.502	.288	3.044	.081	1.652		
	$IAT_{candidates}$* Decidedness	.177	.324	.298	.585	1.193		

$Explicit_{candidates}$	.185	.289	.410	.522	1.203
$Explicit_{party\text{-}based}$	3.590	.386	86.443	< .001	36.224
$Explicit_{candidates}$* Decidedness	.561	.396	2.010	.156	1.752
$IAT_{candidates}$* $Explicit_{candidates}$	.071	.215	.108	.742	1.073

Note. *N* = 620. B: regression weight B; *SE*: standard error of the regression weight B; Wald: Wald criterion; Exp(B): Odds ratio. Relative amount by which the odds increase (Exp(B) > 1.0) or decrease (Exp(B) < 1.0) when the value of the predictor is increased by 1 unit; CCC: correctly classified cases; DV: voting behavior (0 = right political camp, 1 = left political camp). All continuous variables were z-standardized prior to the analyses.
